# Comparison of Drip, Pipe and Surge Spring Root Irrigation for Jujube (*Ziziphus jujuba* Mill.) Fruit Quality in the Loess Plateau of China

**DOI:** 10.1371/journal.pone.0088912

**Published:** 2014-02-14

**Authors:** Qing-Han Gao, Jin-Gang Yu, Chun-Sen Wu, Zhi-Sheng Wang, You-Ke Wang, De-Lan Zhu, Min Wang

**Affiliations:** 1 College of Food Science and Engineering, Northwest A&F University, YangLing, Shaanxi, China; 2 School of Public Health, Ningxia Medical University, Yinchuan, Ningxia, China; 3 Laboratory Animal Center, Ningxia Medical University, Yinchuan, Ningxia, China; 4 College of Resources and Environment, Northwest A&F University, YangLing, Shaanxi, China; 5 College of Water Resources & Architectural Engineering, Northwest A&F University, YangLing, Shaanxi, China; University of Vigo, Spain

## Abstract

Loess Plateau is a typical rain-fed farming region, facing the threat of drought. Irrigation method is among the most important factors affecting jujube quality. This study investigated the response of *Ziziphus jujuba* Mill. cv. Lizao quality to three different irrigation methods (drip-, pipe- and surge spring root irrigation) combining two water levels (20 m^3^/hm^2^ and 120 m^3^/hm^2^). The effects of the trials were evaluated by taking into account the physical-chemical characteristics of jujubes and the antioxidant activity. Concomitant to this, the concentration of some taste-related (viz. glucose, fructose, TSS and malic acid) and health-related compounds/parameters (viz. catechin and epicatechin) were generally much greater in jujube fruit treated with drip irrigation (120 m^3^/hm^2^). Different irrigation treatments had no significant effects on antioxidant capacity, total phenolics and proanthocyanidins (except for pipe irrigation 20 m^3^/hm^2^). The best compromise between quality and irrigation of jujube fruit was achieved with drip irrigation (120 m^3^/hm^2^).

## Introduction

Fruits are good sources of natural antioxidants and biologically active components, and play an important role in human nutrition in supplying certain constituents in which other food materials are deficient [1]. In particular, jujube fruit is considered as a functional food, due to the epidemiological evidence that a high consumption of jujube, and of all its industrial products, is correlated with a reduced risk of some types of cancers [2], [3]. Jujube is recommended for the treatment of some diseases like cardiovascular disease related to the production of radical species resulting from oxidative stress [4]. The contributory factors are due to the presence of vitamins and provitamins, such as ascorbic acid, tocopherols and carotenoids. Additionally, they are rich in a wide variety of phenolics with a high oxygen-radical scavenging and quenching capacity [5], [6].

However, the nutrient content of jujube fruit mostly depends on genetic and environmental factors, and the ripening stage [7]. Agricultural practices, such as irrigation, can also influence the nutrient content in fruit [8], [9]. Several authors reported that deficit irrigation improved peach fruit quality without affecting tree productivity [10].

**Figure 1:**
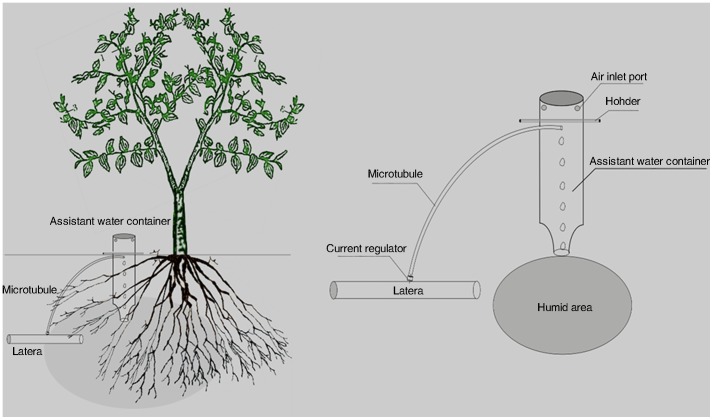
Surge spring root irrigation for jujube trees in loess plateau region part of China.

Jujube (*Ziziphus jujube* Mill.) is one of the most common and economically important fruit tree species in the Loess Plateau area, where drought periods are frequent and water resource is the major factor limiting irrigated agriculture. The loess plateau growers are facing increasing pressure to reduce water use by improving water management. In view of the water crises in the arid area, the loess plateau of China has been tenaciously following the strategy of preserving its water resource and protecting the greenery there. The program reduces water consumption through applying the optional irrigation method. Modern irrigation systems such as surge spring root irrigation and drip irrigation techniques seemed to be promising for use in the arid and semi-arid areas, since water usage is more efficiently, and the small output might prevent a sudden rise in the water table, as compared with other irrigation methods, such as basin or sprinkle irrigation. Thus, it is anticipated that a considerable amount of water will be saved by converting the traditional irrigation systems into the modern methods. Even jujubes would grow and yield well, however, only a part of the entire space between trees is wetted [11]. The aim of the present study was to analyze of the quality changes of jujube fruit produced by different irrigation practices (drip irrigation (DI), pipe irrigation (PI) and surge spring root irrigation SSRI (Figure 1). The effects of the trials were evaluated by taking into account the physical and chemical characteristics of the fruits, as well as the antioxidant activity.

## Materials and Methods

### Chemicals

Pure standards of succinic acid, malic acid, citric acid, fructose, glucose, sucrose, catechin, epicatechin, cinnamic acid, rutin, quercetin, Folin–Ciocalteau reagent, 2, 2′-azinobis(3-ethylbenzothiazoline-6-sulphonic acid) diammonium salt (ABTS), 2, 2-diphenyl-1-picrylhydrazyl (DPPH), 6-hydroxy-2,5,7,8-tetramethylchroman-2-carboxylic acid (Trolox) were purchased from Sigma Chemical Co. (St. Louis, MO). Other reagents were of analytical grade.

### Plant Material and Experimental Design

#### Site description

Field experiments were conducted in a jujube orchard at Yulin Agro-Eco Experimental Station, which is a research center of Northwest A&F University, and the field studies did not involve endangered or protected species. The Agro-Eco Experimental Station is located in Shaanxi Province in the North China (110°17′E, 37°36′N; average elevation, 1049 m). This area has a semi-arid continental climate (based on data for 1956–2006): with mean annual precipitation of 505 mm; a mean annual temperature of 8.6°C, with mean monthly temperatures ranging from −6.5°C in January to 22.8°C in July; 2720 h of sunshine on average each year [12]. The whole area of the orchard is covered by loess (Inceptisols, USDA) with silt loam texture, which developed from wind-deposited loess parent material [13].

#### Treatments

The experimental design was randomized complete block with three replicates per treatment. Each treatment contained four rows with five trees in each row. The seven treatments were the control treatment (CK) involved no irrigation over the entire crop season, DI, PI and SSRI. Each irrigation treatment was applied with two levels of water (20 m^3^/hm^2^ or 120 m^3^/hm^2^) per tree. Each irrigation treatment separated from each other with at least a row of none experimental trees.

All of the treatments were managed identically, and typical pruning and pest and disease control were applied to the trees. Weed control was performed manually. The jujube trees were irrigated for two times (applied at blossoming and bearing fruits stage on 21/5/2010 and 22/7/2010 fruit spreading growth stage, respectively) over the entire crop season.

#### Samples

A total of 1 kg of jujube fruits picked from eight year old jujube trees at their white ripening stage was subjected to each irrigation treatment with three replicates.

### Quality Indexes

The moisture content of jujube fruits was determined by an oven-drying method at 70°C until constant weight was achieved. The moisture content of the fruit was calculated as a percentage loss of the fruit weight. The content of total soluble solids (TSS) in the juice was measured with a refractometer (Atago Co. Ltd., Tokyo, Japan) and titratable acidity (TA) was determined by titration with NaOH and phenolphthalein indicator. Ascorbic acid content was determined using the 2, 6-dichlorophenolindophenol titration method [14].

### Sugar and Organic Acid Determination

For the extractions of sugar and organic acid profiles, 5 g of fruit was extracted with 50 mL of purified water by ultrasonic bath for 20 min. The supernatant was separated and the residue was re-extracted by repeating the above steps under the same conditions. The two filtrates were combined, and then the solvent was evaporated under vacuum at 65°C for sugars and 55°C for organic acids. All extracts were stored at −20°C in the dark until use. Samples were filtered through a 0.45 µm membrane filter (Iwaki Glass) before HPLC analysis.

The concentrations of soluble sugars (glucose, fructose and sucrose) in the fruit were determined by a Waters HPLC system with a refractive index detector (Waters corp., USA) [8]. The HPLC analysis was performed using an Inertsil NH_2_ column (4.6 mm×250 mm, 5 µm) (GL Sciences, Japan). The mobile phase was acetonitrile: water (80∶20), with a flow rate of 1.4 mL/min. The column was operated at 35°C. Sample injection volume was 10 µL.

The organic acid concentration was determined by Waters HPLC system (Waters corp., USA) based on the method described by Gao et al. [6], using an Atlantis T_3_ column (4.6×150 mm, 3 µm) (Waters corp., USA), coupled with a 2487 UV–Vis wavelength detector set at 210 nm. The mobile phase was 0.5% NH_4_H_2_PO_4_, adjusted to pH 2.6 with ortho-phosphoric acid. The flow rate was 0.8 mL/min.

### Phenolic Compounds Determination

20 g of jujubes were blended for 3 min in 200 mL of 80% methanol using a Waring blender. The mixture was then homogenized in a high-speed homogenizer for 3 min and then placed in an ultrasonic bath and sonicated for 20 min. Samples were then filtered through a 0.45 µm microporous membrane. The filtrate was collected, and the solid was extracted two more times with the same volume of fresh solvent. The two filtrates were combined and filtered in a vacuum and rinsed with 100% methanol, and then the solvent was evaporated using a rotary evaporator at 45°C until the weight of the evaporated filtrate was <10% of the weight of the original filtrate. The final evaporated filtrate were collected carefully into a volumetric flask and standardized to a final volume of 25 mL with methanol. All extracts were stored at −20°C in the dark until use.

The extracts were analyzed using an analytical HPLC unit (Waters), with a Dikma Diamonsil C18 column (4.6×150 mm, 5 µm) (Dikma Technologies Inc., USA). The solvent system used were methanol (A) and ultrapure water (pH 2.6) (B). Elution was performed at a flow rate of 0.8 mL/min and the gradient was as follows: 15% A at 0 min, 25% A at 15–25 min, 75% A at 65 min, 15% A at 70 min. Detection was achieved with a binary pump UV-Vis detector (2487). The compounds in each sample were identified by comparing their retention times with the standards and quantified of catechin, epicatechin, rutin, cinnamic acid and quercetin at 280 nm.

### Total Phenolics Content (TPC) Determination

For total phenolics determination, the samples obtained for individual phenolic compounds analysis were used. TPC of jujube fruit extracts was determined using the Folin–Ciocalteu reagent [15]. The reaction mixture contained 125 µL of extract, 500 µL of deionized water and 125 µL of Folin-Ciocalteu reagent. The mixture was allowed to react for 6 min then 1.25 mL of Na_2_CO_3_ (7%) solution and 1 mL distilled water were added and mixed well. The solution was incubated at room temperature in the dark for 1.5 h. The absorbance was measured at 760 nm using a spectrophotometer. Gallic acid was used as a standard and results were calculated as milligram of gallic acid equivalents (GAE) per 100 gram of extract.

### Proanthocyanidin Determination

For proanthocyanidin determination, the samples obtained for total phenolics analysis were used. Proanthocyanidin content was determined in sealed tubes, 0.1 mL sample was added to a mixture of 0.9 mL MeOH, 6 mL *n*-BuOH/concentrated HCl (95∶5 v/v) and 0.2 mL of a 2% NH_4_Fe(SO_4_)_2_·12 H_2_O solution in 2 M HCl. Absorbance was read at 550 nm before and after heating for 40 min at 95.0±0.2°C [16].

### Antioxidant Activity Determination

The tests used to determine the antioxidative capacity of the fruit were the ABTS and DPPH radical scavenging assay. The antioxidant activity was expressed as mmol Trolox eq./100 g FW.

ABTS radical cation scavenging assay was carried out following a modified method of Iqbal et al [17]. ABTS radical cation was prepared by passing a ABTS aqueous solution through the oxidizing reagent, manganese dioxide, on Fisher Brand P8 filter paper. Excess manganese dioxide was removed from the filtrate by passing the solution through a 0.2 mm Fisher Brand membrane. The extracts were diluted with phosphate buffered saline (PBS, pH 7.4), to an absorbance of about 0.700 (±0.020) at 734 nm. 200 µL of each of the extracts was added to 3 mL of diluted ABTS**^·^** ^+^ solution and the absorbance reading was taken 1 min after the initial mixing at room temperature. PBS was used as the blank.

DPPH radical scavenging capacity was estimated according to He et al. with slight modification [18]. One milliliter of diluted extract was mixed with 1 mL of DPPH solution, the mixture was kept in the dark for 30 min and the absorbance at 517 nm was measured.

### Statistical Analysis

The data were analyzed using SPSS software (PASWStatistics18.0). All results are expressed as the mean ± standard deviation (SD) of three replicates. Two-way analysis of variance (ANOVA) was used to evaluate differences between treatments. All of the statistical differences were carried out at a significance level of *α* = 0.05.

## Results and Discussion

### Quality Indexes

The quality characteristics of jujubes including fruit weight, moisture content, TSS, TA and ascorbic acid of jujubes cultivated under SSRI, PI and DI treatments at two water levels are shown in Table 1. DI 120 was positively influenced the quality characteristics of jujube fruits when compared to other irrigation treatments.

**Table 1:** Moisture, fruit weight, total soluble solids (TSS) and titratable acidity (TA) of jujube fruit, in relation to the different Irrigation practices.

Compounds	Control	SSRI (m^3^/hm^2^)		DI (m^3^/hm^2^)		PI (m^3^/hm^2^)	
		20	120	20	120	20	120
Moisture (%)	19.2±0.1 cd	17.3±0.1e	18.5±0.3 d	20.1±0.2 bc	21.3±0.2 a	19.2±0.1 cd	20.5±0.3 ab
Weight (g)	16.7±0.4 e	22.9±1.5 d	26.7±1.0 c	23.4±0.7 d	31.8±1.3 a	25.7±1.1 c	28.9±0.2 b
TSS (%)	15.3±0.2 c	12.9±0.1 d e	14.3±0.2 d	16.1±0.4 b	17.1±0.1 a	15.0±0.1 c	17.2±0.1 a
TA (%)	0.07±0.01 bc	0.07±0.01 c	0.11±0.01 a	0.06±0.01 c	0.10±0.02 ab	0.10±0.0 ab	0.13±0.03 a

Data are mean ± standard deviation for n = 3. Means in the same line with different letter are significantly different (*P*<0.05).

Fresh fruit weight is an important external quality attribute of jujube fruit and is mainly determined by the cultivar. However, within the same cultivar, the fresh weight is also affected by the irrigation treatment to some extent [19]. Fresh jujube fruit weight and moisture content of DI 120 generally had higher values than those of SSRI and PI. The values of fruit weight and moisture content increased with increasing applied water level (Table 1), meaning that well-irrigated trees increased the size of jujube fruit. In addition, fruit weight increases in the drip-irrigation could be attributed in part to increased nutrient availability under irrigation, as indicated by Liu et al [20]. A previous study has indicated that fresh weight of full irrigation fruits was higher in low crop load than in commercial crop load because of the higher water content [21].

In jujube, TSS at the ripe stage is the most important factor for consumer acceptance regardless of acidity. Analysis of fruit quality data indicated a significant increase in fruit TSS content in DI (20 and 120 m^3^/hm^2^) and PI (120 m^3^/hm^2^) treatments. Jujube fruits under the treatments of DI (120 m^3^/hm^2^) and PI (120 m^3^/hm^2^) reached higher values of juice TSS and TA than control fruits, and they could have improved the flavour of the fruit juice and therefore the commercial quality and consumer acceptance. The present results were in accordance with those obtained by Malash et al [22], working on tomato who found that fruit weight, number, and TSS were increased under trickle method compared with furrow method. Moreover, Amer [23] reported interaction between season and irrigation quantity significantly affected TSS and fruit weight. Titrable acidity was positively correlated with the irrigation volume. Our results were in agreement with Favati, et al [24]. Salinity has been reported to improve fruit quality by increasing TSS and TA concentrations [25]. Drip irrigation in the greenhouse had been described as a cause for the increased content of TSS in tomato [26]. In drip irrigation when water was applied in lesser amount, sugar imported by fruits via phloem become concentrated which help in increasing TSS content and pH of tomato [26]. Apricots from deficit-irrigated trees had higher TSS, TA, and hue angle than control fruit and similar fruit diameter, fresh mass, flesh firmness, and maturity index [27]. Deficit irrigation application in stage II of fruit growth, in a mid- to late-maturing peach cultivar, resulted in higher TSS, lower TA, and higher TSS/TA ratio compared to those in control fruit [28].

It has long been recognized that ascorbic acid has a unique and vital beneficial role in the human diet and the juice of jujube fruit provides an important source of ascorbic acid for human nutrition. In the present work, No significant difference in the ascorbic acid content was shown for the seven treatments (Table 2). Drip irrigation in the greenhouse caused an increase in ascorbic acid content of tomato by 85.9% over the outdoor surface irrigated crop due to lesser amount of water available to fruits at shorter interval which caused osmotic adjustment in the pericarp of tomato and resulted in higher ascorbic acid content and pH [29].

**Table 2:** Effect of different irrigation methods on ascorbic acid (mg/100 g FW), total phenolics (mg GAE/100 g FW), proanthocyanidins (mg GSPE eq./100 g FW), DPPH (%) and ABTS (mmol trolox eq/100 g FW) in jujube fruits.

Compounds	Control	SSRI (m^3^/hm^2^)		DI (m^3^/hm^2^)		PI (m^3^/hm^2^)	
		20	120	20	120	20	120
Ascorbic acid	208.9±15.1 abc	176.0±6.9 d	225.0±15.9 a	203.1±9.1 abcd	222.0±26.9 ab	188.9±12.0 cd	194.8±7.9 bcd
Total phenolics	396.2±35.9 b	398.1±27.4 b	430.5±36.1 ab	438.0±10.6 ab	458.2±17.7 a	395.2±6.8 b	440.3±11.1 ab
Proanthocyanidins	434.8±10.4 b	491.0±35.1 a	479.3±52.4 ab	486.1±20.4 ab	482.6±5.4 ab	386.0±24.2 c	457.1±17.4 ab
Antioxidant activity							
DPPH	17.0±5.4 c	25.5±5.0 ab	28.5±5.5 a	19.2±2.8 bc	23.4±2.2 abc	24.0±0.6 abc	28.9±3.9 a
ABTS	3.4±0.2 c	4.4±0.2 abc	5.4±1.3 a	3.9±0.3 bc	3.8±0.4 bc	4.3±0.8 abc	5.1±0.5 ab

Data are mean ± standard deviation for n = 3. Means in the same line with different letter are significantly different (*P*<0.05).

### Proanthocyanidins

There was no significant difference in the proanthocyanidins content in the control and irrigation treated samples except for PI 20. Our results agree with a previous study in which red wine-grapes treated with an early water stress is linked to little impact on the content of proanthocyanidins and other flavonoids [30]. In peaches, regulated deficit irrigation increased fruit phenolic content, especially anthocyanin and proanthocyanidin content [21].

### Total Phenolics and Antioxidant Activity

No statistically significant differences were detected among concentrations of TPC with different irrigation treatments (Table 2). Results proved that different irrigation treatments applied to jujube trees did not significantly affected the TPC present in jujube samples, but significant differences were observed with their HPLC profiles. Jordán, et al. [31] also reported that no statistically significant differences were detected on concentrations of phenolic components among the values of the four watering level treatments studied. Our results are not in agreement with previous results on phenolic concentrations in olive oils, in which they reported that TPC were higher in oils from non-irrigated trees [32]. It may well reflect varietal differences as well as the exact agronomic conditions. In olive fruit, grown under different irrigation regimes, higher TPC was measured with decreased irrigation water volume [33]. Navarro et al. [8] reported that the juice of Clemenules mandarin fruit had a significantly increased level of TPC when deficit irrigation treatments were applied.

The antioxidant activity of fruits is important for assessing their nutritional value. The antioxidant activities of the different extracts, according to the irrigation treatments applied, are shown in Table 2. As reported for TPC, no statistically significant differences were found for the radical-scavenging activity of the jujube sample extracts. TPC seems to be a good indicator of the antioxidant potential in jujube fruit. Pernice et al. [34] also found that no significant differences for the radical-scavenging activity exist between the irrigated samples and the control.

### Sugar, Organic Acid, Phenolic Compositions

Sugars are the major component of TSS apart from organic acids, amino acids and soluble pectins. Sweetness which is related to the sugar content of the fruit is an overriding factor in the eating quality of jujubes. Though the consumer preference varied but in general, consumer prefers fruit with high sugar and low acid levels [35]. Apart from the genetics of a cultivar, fruit quality is influenced by cultural practices such as nutrition, irrigation and harvesting [35], [36].

Sucrose was the main soluble sugar found in jujubes whereas fructose and glucose were the main reducing sugars (Table 3). Fructose is present in the largest amounts for jujube. Sugars were differentially affected by irrigation practices. Fructose and glucose were both much higher in fruit from plants treated with DI 120 as compared to other treatments. There was a significant interaction between irrigation and fruit sucrose, where the concentration was greatest in jujube fruit from plants treated with PI 120. DI 120 treatment resulted in highest levels of glucose (2431.7 mg/100 g FW) in jujube fruit. A 5.6-fold increase was observed in glucose levels in DI in comparison to the control. There was a 3.1-fold increase in glucose levels from 20 m^3^/hm^2^ to 120 m^3^/hm^2^ in pipe irrigation in comparison 2.6- to 1.6-fold increase in SSRI and DI, respectively. Similarly, all the 120 m^3^/hm^2^ treatments resulted in higher levels of fructose in the fruit in comparison to the control. Sánchez-Bel et al. [37] reported that the drip irrigation produced almonds with a higher content in sucrose and glucose. Thakur et al. [35] reported that the reduction in the levels of glucose and fructose at harvest in nectarines with deficit irrigation treatments may be ascribed to their higher utilisation in respiration at fruit maturity.

**Table 3:** Effect of different irrigation treatments on the contents of sugar, organic acid, phenolic compositions of jujube fruits.

Compounds	Control	SSRI (m^3^/hm^2^)		DI (m^3^/hm^2^)		PI (m^3^/hm^2^)	
		20	120	20	120	20	120
Sugars (mg/100 g FW)							
Sucrose	nd a	nd	308.4±24.9 b	nd	262.8±19.2 b	nd	418.0±27.9 a
Glucose	436.3±16.4 d	556.0±19.6 d	1447.9±71.2 c	1509.1±95.0 c	2431.7±400.8 a	634.1±48.5 d	1947.0±207.7 b
Fructose	694.5±30.1 e	574.9±26.3 e	1518.4±18.8 d	1836.4±201.9 c	2633.9±164.6 a	711.0±29.9 e	2150.8±178.3 b
Organic acids (mg/100 g FW)							
Citric acid	34.0±2.0 e	66.2±4.5 d	135.7±5.0 b	101.9±4.6 c	138.1±4.9 b	74.7±5.8 d	197.7±32.6 a
Malic acid	98.2±5.8 c	117.9±6.3 c	348.6±36.5 ab	313.5±40.1 b	392.9±29.4 a	99.6±7.2 c	320.5±23.5 b
Succinic acid	17.4±0.7 e	46.2±5.5 cd	74.6±5.2 b	53.8±6.5 c	68.2±5.3 b	38.7±5.3 d	123.1±6.5 a
Phenolic compounds							
Catechin (mg/100 g FW)	5.64±0.58 d	14.9±0.8 b	15.4±0.7 b	13.4±0.4 c	16.5±0.6 a	16.6±0.5 a	15.8±0.4 ab
Epicatechin (mg/100 g FW)	21.2±0.8 d	32.2±1.1 bc	30.4±1.1 c	25.4±0.9 d	37.5±3.1 a	36.7±4.6 ab	37.1±4.5 ab
Rutin (mg/100 g FW)	1.44±0.05 a	1.17±0.05 b	0.96±0.03 d	0.71±0.04 e	1.25±0.06 b	1.06±0.06 c	1.23±0.06 b
Quercetin (µg/100 g FW)	nd	56.0±3.5 a	20.7±2.4 c	22.7±2.2 c	nd	49.7±2.4 b	nd
Cinnamic acid (µg/100 g FW)	nd	10.3±0.4 b	9.9±0.5 bc	nd	nd	11.5±0.6 a	9.2±0.5 c

Data are mean ± standard deviation for n = 3. Means in the same line with different letter are significantly different (*P*<0.05).

a Abbreviations: nd, not detected.

Organic acid content of a fruit is an important factor for the development of its flavour. Organic acids were differentially affected by irrigation methods (Table 3). Citric acid, malic acid and succinic acid were all lower in fruit from plants treated with 20 m^3^/hm^2^ water as compared to 120 m^3^/hm^2^ water for each irrigation method. There was a 4-fold increase in citric acid levels from 34.0 mg/100 g FW (control) to 135.7 mg/100 g FW (SSRI 120 m^3^/hm^2^) in comparison to 4.0 and 5.8-fold increase in DI 120 and PI 120, respectively. A 3-fold increase was observed in malic acid levels in jujube fruits irrigated with 120 m^3^/hm^2^ water in comparison to the control. But the levels of malic acid in the fruit did not differ among each treatment with 120 m^3^/hm^2^ water. The PI 120 treatment resulted in highest succinic acid level (123.1 mg/100 g FW) in jujube fruit. A 4.3-fold increase was observed in succinic acid levels from 17.4 mg/100 g FW (control) to 74.6 123.1 mg/100 g FW (SSRI 120) in comparison to 3.9-fold increase in DI 120. Sánchez-Bel et al. [37] reported that the irrigation produced almonds with a higher content in oxalic, citric and malic acids than the nonirrigated almond trees. Souza et al. [38] observed in grapes an increase of these same organic acids in grapes under irrigation. The effect of water stress during the herbaceous period of berry development on acidity has been earlier reported for other varieties or conditions [35], [36].

Changes in the phenolic compounds of jujubes differed according to the irrigation treatments since fruits are collected in the same date, and cultivated under the same crop conditions (Table 3). Water availability can considerably affect plant phenolic metabolism and composition in fruit [39]. Flavonoids (including epicatechin, catechin, rutin and quercetin) are commonly classified as ‘‘environmental compounds’’ because they are often produced in direct response to environmental conditions [40]. For example, it has been reported that the flavonoids content is dependent on UV-B irradiation, water stress and CO_2_ levels [41], [42]. The obtained results show that water management may influence the individual phenolic content of jujube fruit. In the present work we report that epicatechin, catechin, rutin, quercetin and cinnamic acid were detected in jujube samples. Similar HPLC patterns of epicatechin, catechin, rutin were observed for all irrigated samples however, their contents differed depending on the irrigation treatments. All the irrigation treatments increased the content of epicatechin and catechin compared to control. The results agreed with Sanchez-Rodriguez et al. [40], who reported that *ZarxZar* and *JosxJos* had the highest values of some individual phenolic compounds under well-watered conditions. The content of rutin reduced for all the irrigated jujube samples compared to control. Epicatechin and catechin are the main antioxidants present in the jujube fruits under different irrigation practices. For irrigated samples, jujube trees with DI 120 had higher contents of epicatechin, catechin and rutin (37.5, 16.5 and 1.25 mg/100 g FW, respectively) whereas SSRI 20, showed a significantly higher content of quercetin (56.0 µg/100 g FW). A small number of cinnamic acid was detected in SSRI and PI treatments.

The levels of water did not cause significantly change of epicatechin and catechin for SSRI and PI, while the higher levels of water for DI enhanced the contents of epicatechin and catechin. The content of quercetin for SSRI 20 was about 2.7-fold compared to that of SSRI 120.

Different hypothesis have been developed to explain the differences found in the phenolic compounds of plants under irrigation: the different water content of the fruits that could imply a different solubilization of phenolics which are more soluble in water and a different effectiveness in the release of phenolic compounds during crushing and malaxation linked to polysaccharides of the cell wall, and the water stress suffered by the trees that could imply a greater synthesis of phenolic compounds in the fruit [8].

From the results obtained in this trial, differences in the content of phenolic compounds in jujubes may be the consequence of the water content of the fruits, since phenolics content in jujubes from control treatment were significantly different from that in jujubes from the irrigation treatments. In peach trees, it has been reported that soil moisture stress may be related to the increase in phenolics in fruits [43]. The results showed that it is possible to modulate antioxidant concentration in the fruit using suitable agricultural practices.

## Conclusions

The data resulting from this research work indicate that jujube fruit had a positive response to irrigation practices but this response differed according to the amount of water applied. TPC, ascorbic acid content and antioxidant activity did not show significant variability with irrigation treatments while different irrigation regimes applied affected quality indexes and the composition of sugar, organic acid and phenolics. It is important for growers to understand the balance between enhancing phytochemical content by applied the appropriate irrigation and maintaining good quality of the product. The loess plateau areas also calls for the establishment of a suitable compromise between quality of jujube fruits and water consumption. Then, the selection of an optimal irrigation treatment of DI 120 for jujube cultivar in loess plateau is full of importance. SSRI as a promising technique in the arid and semi-arid areas supplies jujubes with water and nutrients at frequent intervals, permits minimal evaporation in comparison with PI and permits slow rates of water application that help maintain a stable water table. More work is necessary to confirm these data over an even longer term, to determine optimum water levels and timing for best fruit quality, and to further improve methods such as SSRI for monitoring and maintaining water consumption.
